# Effectiveness of two-dose vs. one-dose varicella vaccine in children in Shanghai, China: a prospective cohort study

**DOI:** 10.3389/fpubh.2024.1320407

**Published:** 2024-06-04

**Authors:** Yue Li, Fang Xu, Meiling Liu, Sashuang Teng, Fan Liang, Fei Wang

**Affiliations:** ^1^Department of Immunization Program, Hongkou District Center for Disease Control and Prevention, Shanghai, China; ^2^Hongkou District Center for Disease Control and Prevention, Shanghai, China

**Keywords:** effectiveness, two-dose, varicella vaccine, child, cohort

## Abstract

**Objective:**

Varicella, a highly contagious viral disease caused by the varicella-zoster virus (VZV), affects millions globally, with a higher prevalence among children. After the initial infection, VZV lies dormant in sensory ganglia and has the potential to reactivate much later, causing herpes zoster (HZ). Vaccination is one of the most effective methods to prevent varicella, and the two-dose varicella vaccine (VarV) regimen is widely used around the world. In China, the VarV has been included in the national immunization programme with a recommended single-dose regimen. This study aimed to compare the effectiveness of the two-dose vs. one-dose VarV regimen in children in Shanghai, China.

**Materials and methods:**

A prospective cohort study was conducted in Shanghai, China, from September 2018 to December 2022. The study enrolled children aged 3–18 years who had received either the one-dose, two-dose, or 0-dose VarV regimen. Vaccination history, varicella infection status, and relevant variables, including demographic information (name, date of birth and sex) and medical history (clinical features of varicella and illness duration) were collected through medical record review and parental interviews.

**Results:**

A total of 3,838 children were included in the study, with 407 in the 0-dose regimen group, 2,107 in the one-dose regimen group and 1,324 in the two-dose regimen group. The corresponding incidence density in these groups was 0.13, 0.05 and 0.03 cases per 1,000 person-days, respectively. The adjusted vaccine effectiveness (VE) was 81.7% (95%CI: 59.3–91.8%) for the two-dose regimen and 60.3% (95%CI: 29.3–77.7%) for the one-dose regimen, compared to the 0-dose regimen. The two-dose VarV regimen showed a protective effectiveness of 47.6% (95%CI: 2.5–71.9%) compared to the one-dose VarV regimen.

**Conclusion:**

This study provides evidence supporting the greater effectiveness of the two-dose VarV regimen in preventing varicella infection compared to the one-dose regimen.

## Introduction

Varicella, also known as chickenpox, is an acute and highly contagious disease caused by the varicella-zoster virus (VZV), a member of the alphaherpesvirus family. Following the initial varicella infection, VZV becomes latent in sensory ganglia and can reactivate years or decades later, leading to herpes zoster (HZ) (1). Varicella is characterized by the appearance of vesicular skin rashes, accompanied by fever and malaise (1). The incubation period of varicella is typically between 10 and 21 days after exposure (1). While the majority of varicella cases present with mild to moderate symptoms, such as the characteristic skin rash, more severe complications can occur, including pneumonia, encephalitis, and hepatitis, particularly in cases of secondary infection (2, 3). Outbreaks of varicella often occur in settings with high population density, such as schools (4, 5). Children under the age of 15, particularly those between 1 and 9 years old, have the highest incidence of varicella infection (6).

Varicella vaccine (VarV) is recognized as the most effective intervention for the prevention and control of varicella (7). The one-dose universal varicella vaccination program, launched in the United States in 1995, has significantly curtailed the occurrence of varicella, as well as the associated hospital admissions and fatalities (8). This program has been remarkably effective, preventing in excess of 91 million cases, around 238,000 hospitalizations, and close to 2,000 deaths (8). In China, the one-dose VarV schedule has been licensed since 1998 for active immunization of children aged 1–12 years, although it is not currently included in the China National Immunization Programme. Despite this, approximately 78% of students aged 3–17 years in Shanghai, China, have reported receiving the one-dose VarV (9). A meta-analysis of 42 studies published between 1995 and 2014 demonstrated that the one-dose VarV vaccine had an effectiveness of 81% against all varicella cases and 98% against moderate and severe varicella (10).

Despite the successful implementation of the VarV programme worldwide, varicella outbreaks continue to occur in school settings, even with high coverage of the one-dose VarV (11). In China, where most children have received one dose of VarV, outbreaks of varicella still occur in primary and middle schools (12). The World Health Organization (WHO) has suggested that this may be due to waning immunity or secondary vaccine failure (11). In Shanghai, China, VarV has been included in the Immunization Programme since August 2018, with children in the city receiving one free dose of VarV at 12 months and one at 4 years of age. Children born on or after August 1, 2014 are eligible for a free second dose of VarV after 4 years of age, while children born before August 1, 2014 may receive a second dose of VarV at their own expense. However, it is still unclear whether this strategy effectively prevents varicella cases. Therefore, we conducted this prospective cohort study to evaluate the effectiveness of a two-dose VarV regimen in children.

## Materials and methods

### Study design and subjects

This prospective cohort study was conducted in the Hongkou District of Shanghai, China. The study protocol was approved by the Ethics Committee of the Hongkou Center for Disease Control and Prevention (Grant number: HKCDC2018-01). The informed consent was obtained from all study participants or their legal representatives (parents or guardians). The study enrolled healthy children between the ages of 3 and 18 years, with a gender distribution of 51.15% male and 48.85% female. Children were excluded from the study if they met any of the following criteria: (1) a history of varicella infection; (2) known allergies to the vaccine or its components, as well as antibiotics; (3) a history of severe adverse events associated with vaccination; (4) currently experiencing acute illness, severe chronic diseases, or acute episodes of chronic diseases; (5) immunodeficiency or undergoing immunosuppressive therapy; (6) parents or legal representatives unable to comply with the requirements of the study protocol. During the period from 2018 to 2022, two domestically produced VarV- Baike (Changchun Baike Biotechnology Co.) and Shanghai (Shanghai Institute of Biological products co., Ltd.)-were administered in the Hongkou district of Shanghai, China. Each of these vaccines featured a similar concentration of the Oka strain VZV, with over 2000 plaque formation units (PFU) in each 0.5 mL dose. Additionally, all of these vaccines required a cold chain storage and transportation temperature maintained between 2 and 8°C.

### Study definition

This study was conducted from September 2018 to December 2022, during which cases of varicella that occurred within this period were recorded. A clinical diagnosis of varicella was made based on the presence of a characteristic pruritic maculopapular vesicular rash without any other identifiable cause. The students participating in the study were divided into three groups based on their varicella vaccination status: unvaccinated, one-dose vaccinated, and two-dose vaccinated. The effectiveness of the VarV was assessed starting from day 31 after vaccination and continued for a duration of 6–7 months for each subject.

### Data collection

The investigation of varicella cases and data collection was carried out by staff at the local center for community immunization and healthcare professionals in the school. Additionally, staff at the local community health center (CHC) actively monitored the China Information System for Disease Control and Prevention to identify any new reported cases by physicians. All the staff involved in the investigation underwent annual training to ensure data quality control. The diagnosis and confirmation of varicella cases were conducted by doctors from tertiary and regional hospitals. The survey was primarily conducted by the physicians whom the students consulted and the CHC physicians. When a student visited a hospital, the attending physician diagnosed and confirmed the case of varicella. Subsequently, the diagnosis was reported by the parents to the healthcare professionals in the school, who then communicated it to the CHC physician. The CHC physician conducted an epidemiological examination of the student based on this information. A standardized surveillance sheet was utilized to collect information on the cases, including varicella vaccination status, clinical symptoms, and prior varicella history. Phone interviews were conducted by staff at the local community immunization centers. The severity of varicella was categorized based on the number of skin lesions, classified as mild (<50 lesions), moderate (50–500 lesions), and severe (>500 lesions or the presence of complications or hospitalization).

### Statistical analysis

Statistical analyses were conducted using IBM SPSS Statistics for Windows, version 12.0 (IBM Corp., Armonk, NY, United States). Descriptive statistics were presented as means ± standard deviations or as percentage frequencies, depending on the nature of the data. For categorical variables, statistical significance between groups was evaluated using Pearson’s Chi-square test, Fisher’s exact test, or the Wilcoxon rank-sum test, as appropriate. For numerical variables, differences were assessed using ANOVA, Kruskal-Wallis, or the Mann–Whitney U test to determine statistical significance. In the logistic regression model, we calculated the outcome odds ratio (OR) in 1-dose vs. 0-dose, or 2-dose vs. 0-dose, or 2-dose vs. 1-dose and estimated VE by the following formula: VE = (1- OR) × 100(%). In multivariate models, we controlled for sex and age. All test statistics and corresponding *p*-values were analyzed using two-sided tests, and statistical significance was defined as *p* < 0.05.

## Results

### Study setting and study participants

**Figure 1:**
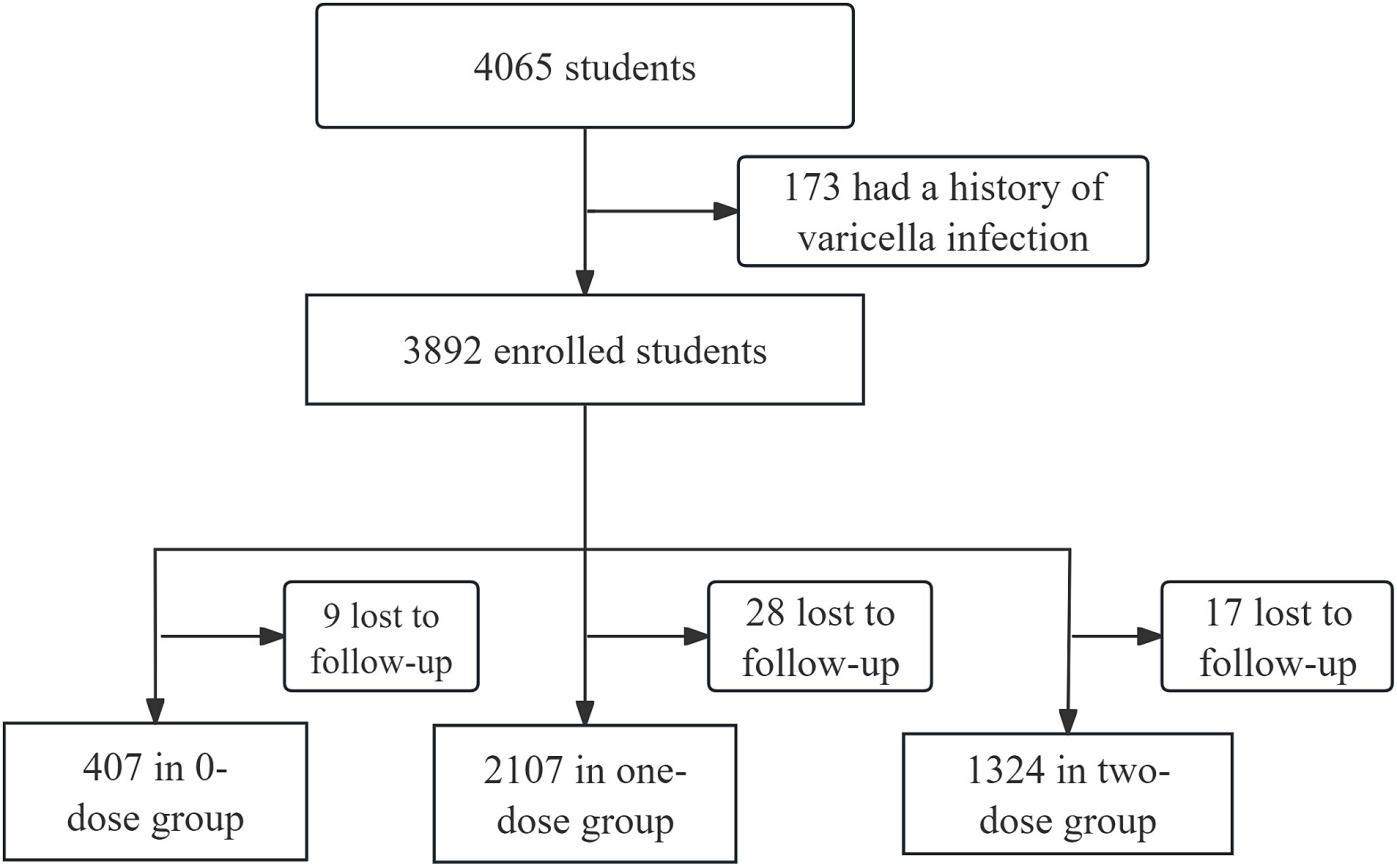
Study flowchart.

Hongkou District, a municipal district located in the northeastern part of downtown Shanghai, accommodates about 650,000 individuals residing across eight streets. There are 8 CHCs providing varicella vaccination services. Out of the initial 4,065 children who underwent eligibility screening, 173 children were excluded due to a history of varicella infection. A total of 3,892 students were included, of whom 416 received 0 dose of VarV, 2,135 received one dose of VarV, and 1,341 received two dose of VarV. During the study period, 54 children (9 in 0-dose group, 28 in one-dose group and 17 in two-dose group) were lost to follow-up due to school transfers or inability to provide information. Therefore, 3,838 students with valid follow-up data were included in the analysis (Figure 1). All vaccinated children had received their first dose of VarV at or after the recommended age of 12 months. The mean age of children in the 0-dose, one-dose, and two-dose groups was 10.15 ± 3.21, 8.32 ± 4.67, and 6.65 ± 4.35, respectively (Table 1).

**Table 1:** Baseline characteristics of study subjects.

Characteristic	0-dose regimen	1-dose regimen	2-dose regimen	*p*-value
No. of subjects	407	2,107	1,324	
Male sex	214(52.58%)	1,075(51.0%)	674(50.91%)	0.828
Age (years)	10.15 ± 3.21	8.32 ± 4.67	6.65 ± 4.35	<0.01
Age strata (year)				
3–5	31(7.62%)	282(13.38%)	411(31.04%)	<0.01
6–11	171(42.01%)	1,119(53.11%)	784(59.21%)	
12–15	75(18.43%)	405(19.22%)	86(6.50%)	
16–18	130(31.94%)	301(14.29%)	43(3.25%)	

### Clinical characteristics of varicella cases

In the 75 reported varicella cases, the majority exhibited mild to moderate clinical presentations (Table 2). Notably, the duration of rash was significantly prolonged in the 0-dose group compared to both the one-dose and two-dose groups (*p* < 0.001). Within the 0-dose vaccine group, 8 cases (42.11%) were accompanied by fever. Conversely, the occurrence of fever was reported in 14 cases (33.33%) in the one-dose vaccine group and 2 cases (14.29%) in the two-dose vaccine group. However, no statistically significant difference in fever incidence was observed among these groups.

**Table 2:** Clinical characteristics of varicella cases post-vaccination during the follow-up.

Variable	0-dose regimen	1-dose regimen	2-dose regimen	*p*-value^a^	*p*-value^b^	*p*-value^c^
No. of varicella cases	19	42	14	–	–	–
Median days duration of rash (range)	6(4–8)	5(3–7)	4(3–6)	<0.01	<0.01	<0.01
>50 lesions, n (%)	0(0)	0(0)	0(0)	1.0	1.0	1.0
Fever, n (%)	8(42.11)	14(33.33)	2(14.29)	0.571	0.131	0.305

^a^Comparing 1-dose regimen vs. 0-dose regimen. ^b^Comparing 2-dose regimen vs. 0-dose regimen. ^c^Comparing 2-dose regimen vs. 1-dose regimen.

### Vaccine effectiveness

During the monitoring period, a total of 75 students developed varicella, with 19 being unvaccinated, 42 having received a single dose of VarV, and 14 having received two doses of VarV. The corresponding incidence density in these groups was 0.13, 0.05, and 0.03 cases per 1,000 person-days, respectively. VE was assessed using logistic regression, presented in Table 3. The preliminary analysis revealed that the crude VE for the two-dose regimen was 78.2% (95% CI: 56.1–89.2%), and 58.5% (95% CI: 27.8–76.1%) for the one-dose regimen. When compared directly, the two-dose regimen showed a protective effectiveness of 47.5% over the one-dose regimen (95% CI: 3.4–71.4%). After adjusting for variables such as sex and age, the effectiveness estimates remained consistent. The adjusted VE was 81.7% (95% CI: 59.3–91.8%) for the two-dose regimen and 60.3% (95% CI: 29.3–77.7%) for the one-dose regimen. The adjusted relative VE between the two regimens was 47.6% (95% CI: 2.5–71.9%).

**Table 3:** Efficacy of varicella vaccine.

	No. of students	No. of cases	Incidence density^a^	Crude VE (95%CI)	Adjusted VE (95%CI)^b^
0-dose regimen	407	19	0.13	–	–
One-dose regimen	2,107	42	0.05	58.5 (27.8–76.1)	60.3(29.3–77.7)
Two-dose regimen	1,324	14	0.03	78.2 (56.1–89.2)	81.7(59.3–91.8)
Two-vs. one-dose	–	–	–	47.5 (3.4–71.4)	47.6(2.5–71.9)

VE, vaccine effectiveness; CI, confidence interval. ^a^Incidence density indicates number of cases per 1,000 person-days. ^b^Adjusted variables: sex and age.

## Discussion

The study conducted in Shanghai, China aimed to evaluate the effectiveness of the two-dose VarV in preventing varicella infection and reducing the severity of the disease in children. The results of the study provided valuable insights into the effectiveness of different vaccination regimens. The findings suggested that the two-dose and one-dose VarV were significantly more effective than the 0-dose VarV in preventing varicella infection, with an adjusted VE of 81.7 and 60.3%, respectively. Moreover, the protective effectiveness of two-dose VarV was 47.6% as compared with one-dose VarV.

VarV has been licensed for use as a single dose in China for children aged 1–12 years since 1998. Although it is not part of the China National Immunization Program, a report in 2012 revealed that 78% of students aged 3–17 years in Shanghai, had received one dose of VarV (9). In response to this, the Shanghai Varicella Emergency Vaccination Program was implemented in 2013, recommending the immediate administration of one dose of VarV to immunize all unvaccinated classmates without a history of varicella when two varicella cases are identified within the same school class (9). Since August 1, 2018, VarV has been officially included in the immunization plan of Shanghai. Children residing in Shanghai who meet the appropriate age criteria are administered a single dose of VarV at 12 months and a second dose at 4 years. Subsequently, a limited number of other cities in China have also introduced the inclusion of a two-dose VarV vaccination into their immunization planning projects, provided free of charge.

This investigation evaluated the effectiveness of a two-dose VarV regimen under a new immunization schedule, revealing an adjusted VE of 81.7% for the two-dose regimen and 60.3% for the one-dose regimen. These results are slightly lower compared to previous studies from France (13), the United States (14–17), Spain (18), Germany (19), and Sweden and Norway (20), where two-dose effectiveness ranged from 92.1 to 92.6%, and one-dose effectiveness ranged from 52.8 to 72.3%. In a 10-year, observer-blind trial across 10 European countries, the effectiveness and safety of two varicella vaccines containing the Oka strain were evaluated in 5,803 children aged 12–22 months (20). Randomly assigned, they received either a two-dose tetravalent measles-mumps-rubella-varicella vaccine, a monovalent varicella vaccine, or two doses of measles-mumps-rubella vaccine (20). The two-dose vaccination schedule exhibited greater effectiveness with rates of 92.1% in Norwegian and 92.6% in Swedish children, compared to 72.3% in Norwegian and 52.8% for one-dose in the same countries (20).

The differences between the results of our study in Shanghai and the European study can be attributed to several key factors, including variations in vaccine types, vaccination rates, and the influence of the COVID-19 pandemic. Firstly, the type of vaccine used is a significant factor. Both studies used vaccines based on the Oka strain, but the European study utilized a tetravalent combination vaccine, in contrast to the monovalent live attenuated vaccine used in our study. This difference in formulation could significantly influence the vaccine’s effectiveness, potentially due to varying immune responses elicited by the different vaccine compositions. Secondly, the extent of vaccination coverage is critical to effectiveness (21). European countries with universal or publicly funded varicella vaccination programs have achieved higher vaccination rates, as evidenced in several studies (22). This contrasts with Shanghai, where free two-dose varicella vaccination was only introduced in 2018, possibly leading to lower vaccination rates among children when compared to European countries. The VarV coverage in Shanghai might not yet meet the 80% threshold recommended by the WHO for optimal community protection (23). We hypothesize that as vaccination rates in Shanghai increase, the observed effectiveness of the vaccine is likely to improve, potentially aligning more closely with the European data. Finally, the COVID-19 pandemic’s impact is a crucial consideration. Our study period, from September 2018 to December 2022, overlapped with the pandemic. Quarantine measures and the resulting changes in children’s social interactions could have altered the transmission dynamics of varicella. Such changes might have affected the observed effectiveness of the vaccine, complicating direct comparisons with periods unaffected by such global health emergencies. Further research might consider isolating the impact of these external factors to better understand the vaccine’s true effectiveness.

Our study demonstrated significant effectiveness of both the two-dose and one-dose VarV in preventing varicella infection, with an adjusted VE at 81.7% for the two-dose and 60.3% for the one-dose, compared to the 0-dose VarV. These results surpass the effectiveness observed for VarV as post-exposure prophylaxis (PEP) among children during varicella outbreaks. In a meta-analysis of 15 studies involving 7,470 children, the VE rates were reported as 60% (95%CI: 35, 75%) for two-dose VarV and 43% (95%CI: 27, 55%) for the one-dose VarV used as PEP (23). The study also highlighted the importance of PEP administration timing; within 3 days of exposure, the effectiveness was 80% (95% CI: 68, 88%), but it reduced to 50% (95% CI: 11, 72%) if begun after 3 days (23). The study further explored PEP coverage impact on prevention, revealing that over 80% coverage could prevent 82% of varicella cases (95% CI: 15, 96%), while a maximum of 80% coverage could prevent 65% (95% CI: 50, 76%) (23). These findings confirm the superior effectiveness of the two-dose VarV regimen and suggest that vaccination rates above 80% enhance vaccine effectiveness.

The two-dose VarV has consistently been found to be more effective than the one-dose version, as reported in numerous studies (24–28). Yin et al. (26) conducted a comprehensive search across five databases for articles published between 1995 and 2017, concluding that the two-dose VarV had an effectiveness rate of 79% in randomized controlled trials (95% CI: 56, 90%), 63% in cohort studies (95% CI: 36, 79%), and 81% in case–control studies (95% CI: 65, 90%). Their findings also suggested greater immunogenicity with the two-dose regimen (26). The reported effectiveness of the two-dose VarV, however, varied across studies, with rates ranging from 81.6 to 100% (25, 29, 30), potentially due to differences in sample sizes, research designs, follow-up durations, and vaccination assessment timings. It is important to note that the effectiveness of VarV during varicella outbreaks is generally lower than observed in other studies, possibly because the intense infection conditions in outbreaks can lead to an underestimation of the vaccine’s effectiveness (31). In a meta-analysis of 12 studies with 87,196 subjects, the overall effectiveness of VarV was found to be 90% (95% CI: 69–97%). However, this effectiveness decreased to 87% (95%CI: 76, 93%) in outbreak scenarios, as opposed to 99% (95% CI: 98, 99%) in non-outbreak studies (32).

The findings of this study are particularly relevant for China, where varicella is a significant public health problem, and the disease burden is high (33, 34). The implementation of the two-dose vaccine regimen in the national immunization program could have a substantial impact on reducing the incidence and severity of varicella disease in the country. Moreover, the study found that the two-dose vaccine regimen was also effective in reducing the severity of varicella disease in children who still contracted the disease despite being vaccinated.

One of the strengths of this study is its prospective cohort design, which allowed for the collection of detailed information on vaccine history and varicella infection outcomes. The use of a large sample size and rigorous statistical analysis also enhanced the validity and generalizability of the study findings. However, some limitations of the study should be acknowledged. Firstly, the study population was limited to children in Hongkou district, Shanghai, and the findings may not be generalizable to other populations in China or other countries. Secondly, diagnosis of varicella cases in this study was based on clinical diagnosis, which may introduce diagnostic bias. Thirdly, the varying mean ages among the groups could influence our findings, as age significantly affects susceptibility to varicella and the immune response to vaccination. The 0-dose group, which may consist of older children, could have a higher likelihood of prior exposure to varicella, leading to natural immunity that might obscure the vaccine’s effectiveness. On the other hand, children in the 2-dose group are likely younger and less exposed, making them more vulnerable to varicella and possibly exaggerating the effectiveness of the two-dose regimen. Such age disparities across groups may bias the results. Further studies with larger and more heterogeneous populations and longer follow-up are essential to confirm our findings and to assess immunity persistence. Comparative effectiveness studies between different vaccine brands should also be pursued.

In conclusion, the results of this study support the current recommendation for the use of the two-dose varicella vaccine regimen in children and suggest that this regimen is more effective than the one-dose regimen in preventing varicella infection and reducing the severity of the disease. Further research is needed to assess the long-term effectiveness and safety of the vaccine and its impact on the global burden of varicella disease.

## Data availability statement

The raw data supporting the conclusions of this article will be made available by the authors, without undue reservation.

## Ethics statement

The studies involving humans were approved by the Ethics Committee of the Hongkou Center for Disease Control and Prevention. The studies were conducted in accordance with the local legislation and institutional requirements. Written informed consent for participation in this study was provided by the participants’ legal guardians/next of kin. Written informed consent was obtained from the individual(s), and minor(s)’ legal guardian/next of kin, for the publication of any potentially identifiable images or data included in this article.

## Author contributions

YL: Data curation, Formal analysis, Investigation, Methodology, Software, Supervision, Writing – original draft, Writing – review & editing. FX: Investigation, Writing – original draft. ML: Data curation, Methodology, Writing – original draft. ST: Data curation, Formal analysis, Writing – original draft. FL: Formal analysis, Project administration, Validation, Writing – review & editing. FW: Conceptualization, Writing – original draft, Writing – review & editing.
